# Easily attainable and low immunogenic stem cells from exfoliated deciduous teeth enhanced the *in vivo* bone regeneration ability of gelatin/bioactive glass microsphere composite scaffolds

**DOI:** 10.3389/fbioe.2022.1049626

**Published:** 2022-12-09

**Authors:** Huacui Xiong, Fujian Zhao, Yuqi Peng, Meimei Li, Huanhuan Qiu, Ke Chen

**Affiliations:** Stomatological Hospital, Southern Medical University, Guangzhou, China

**Keywords:** stem cell from exfoliated deciduous teeth, bioactive glass, bone regeneration, osteogenic differentiation, AMPK signaling pathway

## Abstract

Repair of critical-size bone defects remains a considerable challenge in the clinic. The most critical cause for incomplete healing is that osteoprogenitors cannot migrate to the central portion of the defects. Herein, stem cells from exfoliated deciduous teeth (SHED) with the properties of easy attainability and low immunogenicity were loaded into gelatin/bioactive glass (GEL/BGM) scaffolds to construct GEL/BGM + SHED engineering scaffolds. An *in vitro* study showed that BGM could augment the osteogenic differentiation of SHED by activating the AMPK signaling cascade, as confirmed by the elevated expression of osteogenic-related genes, and enhanced ALP activity and mineralization formation in SHED. After implantation in the critical bone defect model, GEL/BGM + SHED scaffolds exhibited low immunogenicity and significantly enhanced new bone formation in the center of the defect. These results indicated that GEL/BGM + SHED scaffolds present a new promising strategy for critical-size bone healing.

## Introduction

Repair of critical-size bone defects remains challenging in the clinic (Huang et al., 2021). Although various bone substitute materials, such as tricalcium phosphate (TCP) (Bohner et al., 2020) and hydroxyapatite (Chakraborty et al., 2022) have been used, large bony defects still cannot be completely repaired (Pare et al., 2020). Even when incorporating specific growth factors or drugs within these materials (Terauchi et al., 2016; Terauchi et al., 2018), it is difficult to achieve efficient bone tissue regeneration because osteogenic-associated stem cells cannot migrate to central part of the critical-size bone defects (Freitas et al., 2019). To address this limitation, the combination of biomaterials and mesenchymal stem cells (MSCs) has become an encouraging strategy (Oryan et al., 2018b; Zhou and Liu, 2022). Loading bone marrow-derived mesenchymal stem cells (BMMSCs) onto scaffolds enhanced healing of bone defects (Kargozar et al., 2017; Chen et al., 2019; Shalumon et al., 2019; Sun et al., 2022). However, limited availability and donor site morbidity restrict the application of BMMSCs. Therefore, alternative sources of stem cells are needed.

Stem cells from human exfoliated deciduous teeth (SHED) have the properties of rapid proliferation and differentiation into different cells, such as odontoblasts, osteoblasts and chondrocytes (Miura et al., 2003; Chen et al., 2014; Su and Pan, 2016; Ko et al., 2020). Compared with other tissues, such as bone marrow and adipose tissue, SHED can be easily obtained with minimum invasiveness (Yuan et al., 2022). In addition, SHED have a stronger proliferative capacity than BMMSs (Kunimatsu et al., 2018). Meanwhile, SHED have outstanding immunomodulatory and immunosuppressive potential (Junior et al., 2020), thus becoming promising candidates for transplantation.

However, simple implantation of stem cells into bone defects seems insufficient because these cells are easily lost and often die. Thus, stem cells have been incorporated into the extracellular matrix (ECM), which can hold the cells together and provide a medium for the cells to interact and migrate (Theocharis et al., 2016; Li et al., 2021). Various types of bone matrix materials, such as gelatin, alginate, and hyaluronic acid, have been applied to repair bone defects (Zhai et al., 2020; Iglesias-Mejuto and Garcia-Gonzalez, 2021; Liu et al., 2021). Among these materials, gelatin largely resembles the natural ECM components (Derkach et al., 2019), which accelerate cell growth and differentiation (Sun et al., 2018; Derkach et al., 2019). However, gelatin has low mechanical strength and cannot release calcium and phosphate ions that are required for bone mineralization, thus limiting its practical applications in bone regeneration.

**FIGURE 1:**
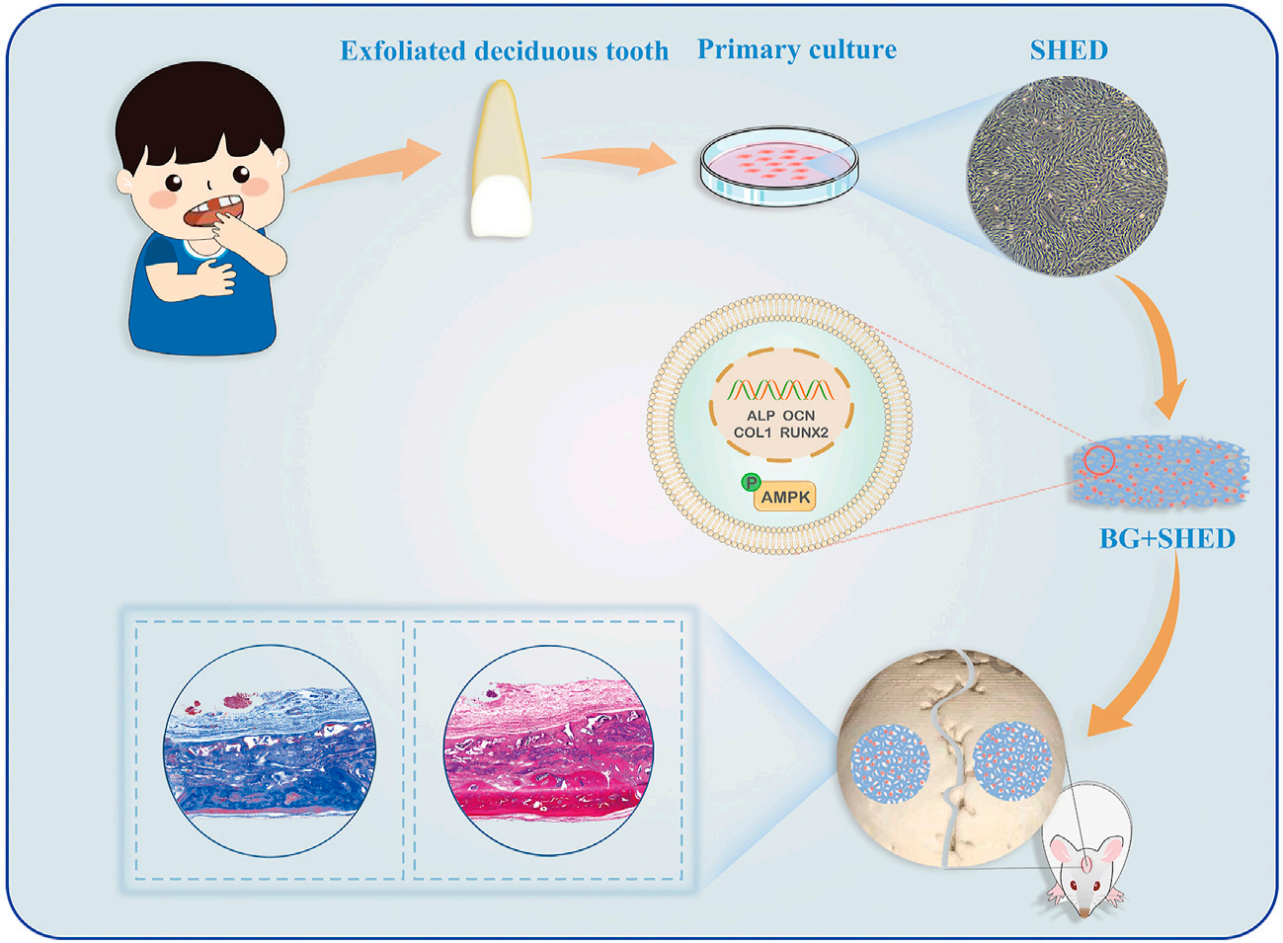
Schematic illustration of GEL/BGM + SHED scaffolds for critical-size bone defect regeneration.

Due to its rapid ion dissolution, bioactive glass (BG) has superior osteoconductive and osteoinductive qualities and thus has been widely used in dentistry and bone repair (Jones, 2013; Zhao et al., 2018b). An apatite layer forming on its surface makes BG bonds to living bones (Hench and Paschall, 1973). We fabricated gelatin/BGM (GEL/BGM) composite scaffolds and described their physicochemical properties in detail in our previous study (Guo et al., 2017; Zhao et al., 2018a). In this study, we loaded SHED into GEL/BGM scaffolds and implanted the cell-scaffold constructs into a rat critical-size bone defect to assess its regeneration ability. We evaluated the potential of BGM to regulate the osteogenic differentiation of SHED. The potential molecular mechanism *in vitro* was also explored (Figure 1).

## Materials and methods

### Synthesis and characterization of bioactive glass microspheres

The synthesis of BGM was conducted based on our previous study (Guo et al., 2017; Zhao et al., 2018a). The molar composition of BGM was 80SiO_2_15CaO5P_2_O_5_. Briefly, a mixture of 25 ml deionized water and 80 ml ethanol was used to dissolve 4 g dodecylamine. After 16 ml ethyl tetraethyl orthosilicate (analytically pure, AR, Guangzhou Chemical), 10.49 ml triethylphosphate (AR, Aladdin) and 24.21 g calcium nitrate tetrahydrate (AR, Guangzhou Chemical) was added to the solution in order, which was then magnetically stirred at 40°C at the speed of 300 rpm. The white precipitate was collected, rinsed and dehydrate at 60°C for 24 h. The next stage was the calcination of the dry precipitate at 650°C for 3 h to obtain BGM. Scanning electron microscopy (SEM, Zeiss Sigma 300, Germany), Fourier transform infrared spectrometry (FTIR, TENSOR27 Bruker, Germany), X-ray diffraction (XRD, ULTIMA IV Rigaku, Japan) and Brunauer-Emmett-Teller (BET, Micromeritics ASAP 2460/2020, United States) were used to examine the morphology and structure of BGM.

### Fabrication of gelatin/BGM scaffolds

The gelatin/BGM scaffolds were fabricated using a freeze-drying method according to our previous report (Guo et al., 2017; Zhao et al., 2018a). Briefly, BGM (28 g) and gelatin (12 g, Aladdin) were dispersed in deionized water (200 ml), followed by stirring for 4 h at 40°C. Three milliliters of genipin solution (1 wt%) was added to the above solution, and the mixture was vigorously stirred for 20 min, frozen for 12 h at −20°C and then freeze-dried for another 24 h. SEM (Zeiss Sigma 300, Germany) and mercury intrusion porosimetry (MIP, Micromeritics AutoPore IV 9500, United States) were used to examine the morphology and porous structure of the scaffolds. To analyze the content of the BGM, thermogravimetric analysis (TGA, Mettler TGA/DSC3+, Switzerland) was used.

### Isolation and cell culture of SHED

Dental pulp tissues were isolated from extracted deciduous incisors (6–8-year-old donors). Parents of these donors signed written informed consent forms. Pulp tissues were isolated, washed, digested in 3 mg/ml collagenase type I (Sigma-Aldrich, United States) for 2 h and incubated with culture medium at 37°C in 5% CO_2_. At 80% confluence, the cells were trypsinized and subcultured. The specific cell surface molecules were identified using flow cytometry. Briefly, cells at passage three were trypsinized and incubated with FITC mouse anti-human CD105 (Cat.561443), PE mouse anti-human CD34 (Cat.555822), PE mouse anti-human CD90 (Cat.555596), PE mouse anti-human HLA-DR (Cat. 555812) and BV510 mouse anti-human CD45(Cat.563204) (all from BD Biosciences, United States) on ice for 30 min. Then the cells were washed, resuspended and detected with a flow cytometry system (BD LSRFortessa, BD Biosciences, Franklin Lakes, NJ, United States).

### Cell attachment and proliferation on GEL/BGM scaffolds

A SHED suspension including 1 × 10^5^ cells was seeded on GEL/BGM scaffolds and incubated for 4 h. Next, normal growth medium was added for further incubation. On day 2, after fixation with 4% paraformaldehyde, the samples were stained with Phalloidin-iFluor 488 Reagent (ab176753, Abcam, United States), and then observed with confocal microscopy (Leica SP8, Germany). On day 7, after fixation with 2.5% glutaraldehyde, the samples were dehydrated, coated with gold and then observed with SEM (Zeiss Sigma 300, Germany). CCk8 assay was used to evaluate the cell proliferation of SHED. GEL/BGM scaffolds were placed into a 48-well plate. 200 μl of cell suspension containing 1 × 10^5^ cells was carefully added to each GEL/BGM scaffold and cultured for 1, 3, and 5 days. Cells without scaffolds were considered as the control group. The medium was changed every day. At each time point, cells were replaced by culture medium with 10% CCK8 ((Dojindo, China) for 4 h. Then, the medium was transferred to a 96-well plate. The optical density (OD) values for each well were measured spectrophotometrically at 450 nm.

### Multipotential differentiation of SHED

SHED at a density of 1 × 10^5^ were seeded into a 12-well plate. After the cells attached to the wall, they were cultured with adipogenic induction liquid (Gibco, United States) or osteogenic induction liquid (Gibco, United States). The cell culture was changed every 3 days. After 3 weeks of induction, the cells were washed and fixed and then stained with 10% Oil Red O staining solution or ARS solution (Sigma-Aldrich).

### Osteogenesis properties evaluation

For osteogenesis property evaluation, BGM extracts were used. The BGM powders were sterilized, added to α-MEM and then kept at 37°C at a shaking speed of 100 rpm for 16 h. After centrifugation, the supernatant was filtered. For further cell culture, BGM extracts were diluted with culture medium or osteogenic medium at a ratio of 1:2.

The osteogenesis effect of BGM extracts on SHED was rated by ALP staining, ARS and osteogenesis-related gene expression. Briefly, SHED were seeded into 12-well plates and incubated for 24 h. Cell medium was substituted with osteogenic medium with BGM extracts and only osteogenic medium, which were considered the BGM and control groups, respectively. In the experiments involving the AMPK inhibitor Compound C (MedChemExpress, United States), 10 μM Compound C was added to the osteogenic medium with BGM extracts. This group was considered the BGM + Com. C group. On day 10, ALP activity was evaluated with NBT/BCIP ALP staining kits (Beyotime Biotechnology, China). After fixation with 4% paraformaldehyde, the cells were cultured with ALP stain working solution (Beyotime) for 1 h. Then, the cultures were washed with PBS and observed under a light microscope. For quantitative analysis, the stained cells were cultured with 100 mg/ml cetylpyridinium chloride (CPC, Sigma-Aldrich) for 1 h and then measured by absorbance at 560 nm. On day 14, cells were stained with 10% ARS solution (Sigma-Aldrich), incubated with 100 mg/ml CPC (Sigma-Aldrich) for 1 h and measured by absorbance at 560 nm.

### Real-time quantitative PCR

TRIzol reagent (Invitrogen) was used to extract RNA. Total RNA was then converted to cDNA *via* HiScript Ⅱ Q RT SuperMix (Vazyme, China). PCR was conducted on a Roche LoghtCycler 96 machine (Roche) with Taq Pro Universal SYBR qPCR Master Mix (Vazyme, China). The reaction conditions were 30 s at 95°C, 40 cycles of 95°C for 10 s and 60°C for 30 s. The sequences of primers for ALP, RUNX2, OCN, and COL1 are shown in Supplementary Table S1 (Supporting Information). The value was calculated *via* the 2^−ΔΔCt^ method and normalized to GAPDH.

### Western blotting analysis

Cells were cultured and treated as described for the ALP assay. On day 4, cells were lysed with RIPA buffer (Beyotime, China). Thirty micrograms of protein was loaded on SDS-PAGE gels and transferred to a PVDF membrane (Millipore, United States). Then, the membranes were blocked and incubated overnight with primary antibodies against AMPKα (CST) and p-AMPKα (Thr172) (CST). After being washed twice with Tris-buffered saline mixed with 0.05% Tween 20 (TBST), the membranes were incubated with a secondary antibody (Proteintech, China). An Enhanced Chemical Luminescence Kit (Forevergen, China) was used to detect the protein bands. Quantitative densitometric analysis was performed using ImageJ software.

### *In vivo* cranial bone regeneration

Twenty-four adult male Sprague Dawley rats (8–10 weeks, 200–250 g) were purchased from the Laboratory Animal Center, Southern Medical University and divided into three groups: the control group (empty defects), GEL/BGM group and GEL/BGM + SHED group. For the GEL/BGM + SHED group, 1 × 10^6^ SHED were loaded on the GEL/BGM scaffolds and subsequently cultured for 4 days prior to implantation into the cranial defects. The GEL/BGM without SHED were also cultured with medium under the same conditions prior to implantation. Pentobarbital (Nembutal, 3.5 mg/100 g) was used for general anesthesia by intraperitoneal injection. The heads of the rats were shaved and disinfected. Then, full-thickness flaps were elevated when incisions were made over the calvarium. A trephine burr was used to create a 5 mm circular defect on each side of the skull. Then, scaffolds were implanted into the defects. The wound was sutured with silk 3–0 suture carefully. Four rats in each group were sacrificed at 4 or 8 weeks after implantation, and their calvarias were immediately excised and fixed in 4% paraformaldehyde.

### Micro-CT analysis and histological assessment

Microcomputed tomography (micro-CT) scanning was performed *via* a Micro-CT (ZKKS-MCT-SharpII, Zhongkekaisheng Co., China) with an operation of 70 kVp voltage and 100 μA electric current. According to the micro-CT results, three-dimensional (3D) images were reconstructed. The reconstructed voxel size was 20 × 20 × 20 μm. After micro-CT scanning, all samples were decalcified using 10% EDTA (pH = 7.4) solution for 4 weeks, embedded in paraffin, and sectioned for H&E and Masson’s trichrome staining. Additionally, immunohistochemistry was performed. The primary antibodies were ALP (affinity, DF12525), COL1a (cloud clone corp), OCN (Proteintech) and RUNX2 (cloud clone corp) and human mitochondria (ab92824).

### Statistical analysis

All results are expressed as the mean ± standard deviation. The statistical significance was determined *via* Student’s t test or one-way analysis of variance. The difference was considered to be statistically significant when *p* < 0.05.

## Results

### Characteristics of SHED

Cells started to grow out from the pulp tissue after approximately 3 days. They presented a spindle and fibroblastic-like morphology (Supplementary Figure S1A). Calcified nodules and intracellular lipid vacuoles were formed in the cultures, as indicators of alizarin red staining (Supplementary Figure S1C) and Oil red O staining (Supplementary Figure S1D). As shown in the flow cytometry analysis, SHED was positive for CD90 (99.75%) and CD105 (99.54%) but negative for CD34 (0.11%), CD45 (0.30%) and HLA-DR (0.09%) (Supplementary Figure S1E).

### Characterization of BGM and cell biocompatibility of GEL/BGM scaffolds

**FIGURE 2:**
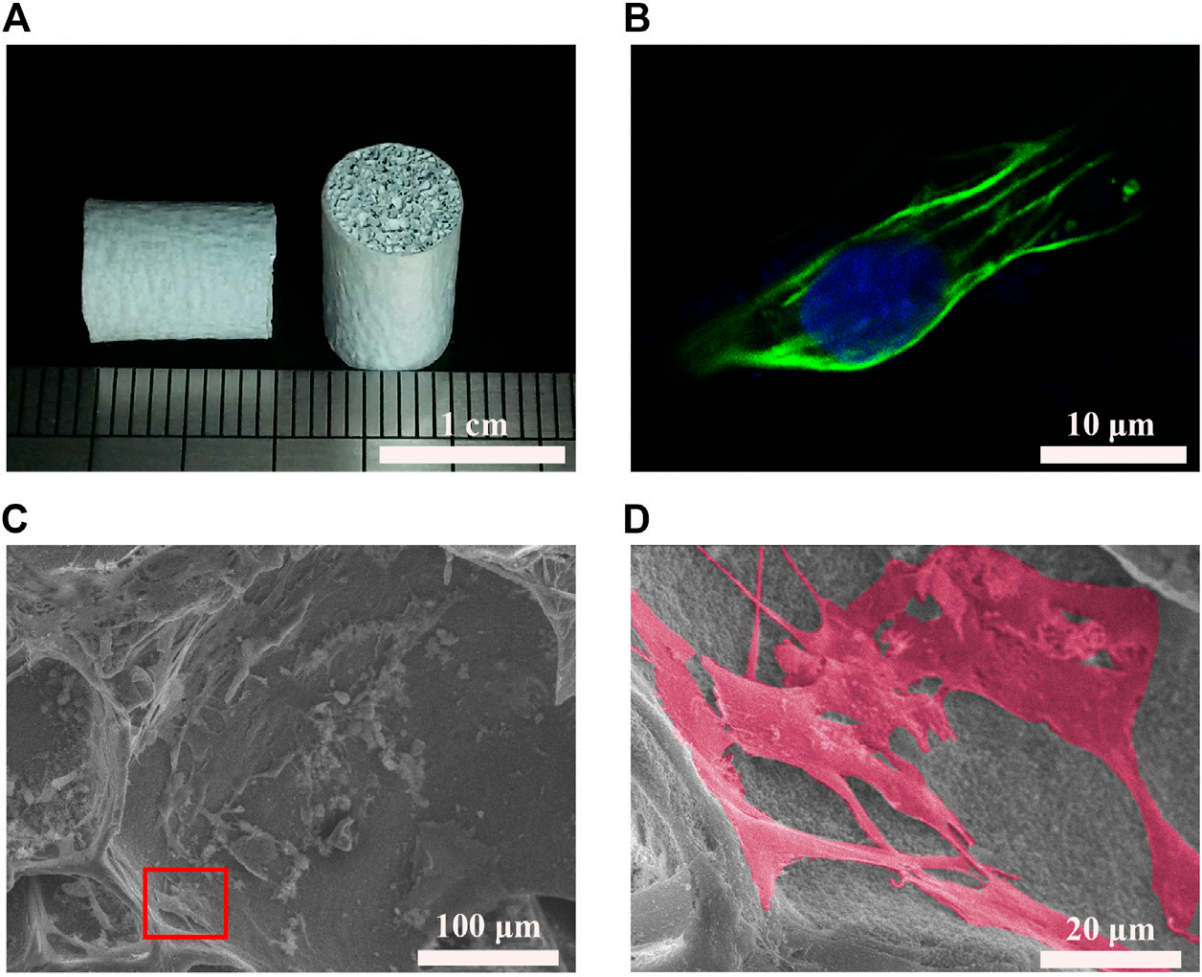
Cell biocompatibility of GEL/BGM scaffolds. **(A)** Gross observation of GEL/BGM scaffolds. **(B)** Fluorescence image of SHED stained with F-actin on day 2. **(C)** SEM image of SHED seeded on the GEL/BGM scaffolds on day 7. **(D)** Magnification of the red box in Panel **(C)**.

The BGMs were spherical with smooth surfaces, and the diameter was approximately 200–400 nm (Supplementary Figures S2A,E). The BET surface area was 14.2504 m^2^/g. The energy dispersive spectrometer (EDS) results indicated the presence of Si, Ca and P in BGM (Supplementary Figure S2B). As shown by the FTIR and XRD results, BGM demonstrated a representative Si-O-Si structure (Supplementary Figures S2C,D). The GEL/BGM scaffolds exhibited a porous structure with a pore size of 200–800 μm (Supplementary Figure S3A). Enlarged images demonstrated that uniform and spherical BG particles were dispersed in the scaffolds (Supplementary Figure S3B). From the TGA results, approximately 68% BGM was added to the composite scaffolds (Supplementary Figure S3C). After culturing for 2 days, the confocal microscopy image demonstrated the presence of well-spread SHED on the scaffolds. Cells presented stretchy morphology (Figure 2B; Supplementary Figure S4A). After culturing for 7 days, SHED attached tightly to the surface of the scaffolds. Cells exhibited a flattened morphology with prominent filopodia (Figures 2C,D). Some cells extended processes to form cytoplasmic elongations and interconnected multicellular networks. In addition, the CCK8 assay showed that SHED proliferated rapidly on scaffolds over time. There was no significant difference between the GEL/BGM and control groups (Supplementary Figure S4B). All results suggested the good cell biocompatibility of the scaffolds.

### Effects of BGM extracts on osteogenic differentiation of SHED

**FIGURE 3:**
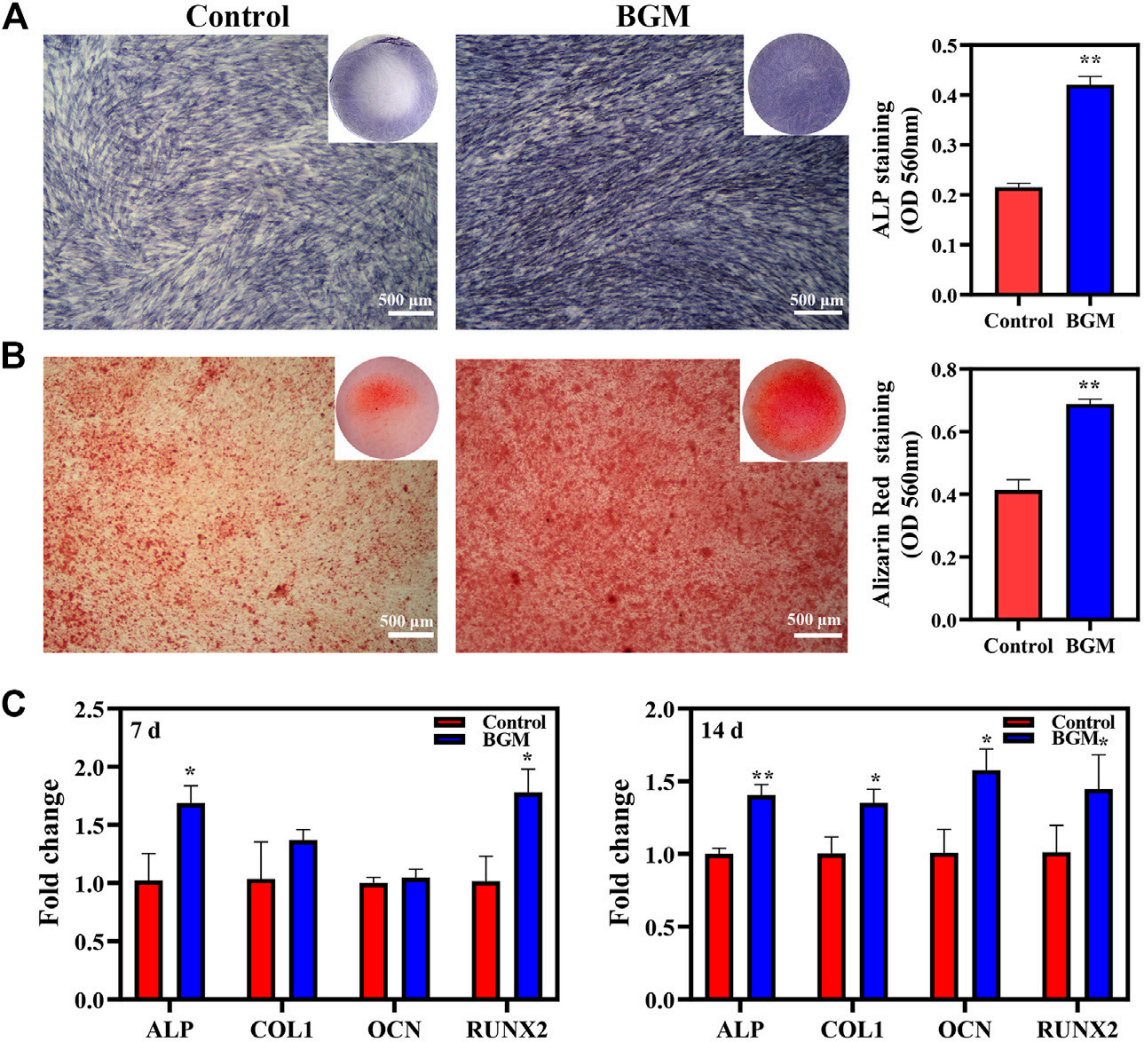
*In vitro* osteogenesis evaluations of SHED stimulated by BGM extracts. **(A)** ALP staining and semiquantification of ALP activity on day 10. **(B)** Alizarin red staining and semi-quantification of mineral deposition on day 14. **(C)** mRNA expression of osteogenesis-related genes (ALP, RUNX2, OCN, COL1) in SHED cultured for 7 and 14 days. *p < 0.05, **p < 0.01 versus the control group.

ALP staining and ARS staining were used to assess the osteogenesis of SHED. On day 10, the BGM group exhibited significantly stronger positive ALP staining than the control group. Quantitative ALP activity showed similar results (Figure 3A). On day 14, positive alizarin red staining indicated that mineralization nodules formed in the culture. Compared with the control group, the BGM group exhibited an increased amount of mineralized matrix (Figure 3B). Additionally, ALP, RUNX2, OCN and COL1 mRNA expression was evaluated. On day 7, the BGM group demonstrated significantly higher ALP and RUNX2 levels than the control group. On day 14, the BGM group showed significantly higher ALP, RUNX2, OCN and COL1 levels than the control group (Figure 3C).

### Mechanism of BGM-stimulated osteogenesis

**FIGURE 4:**
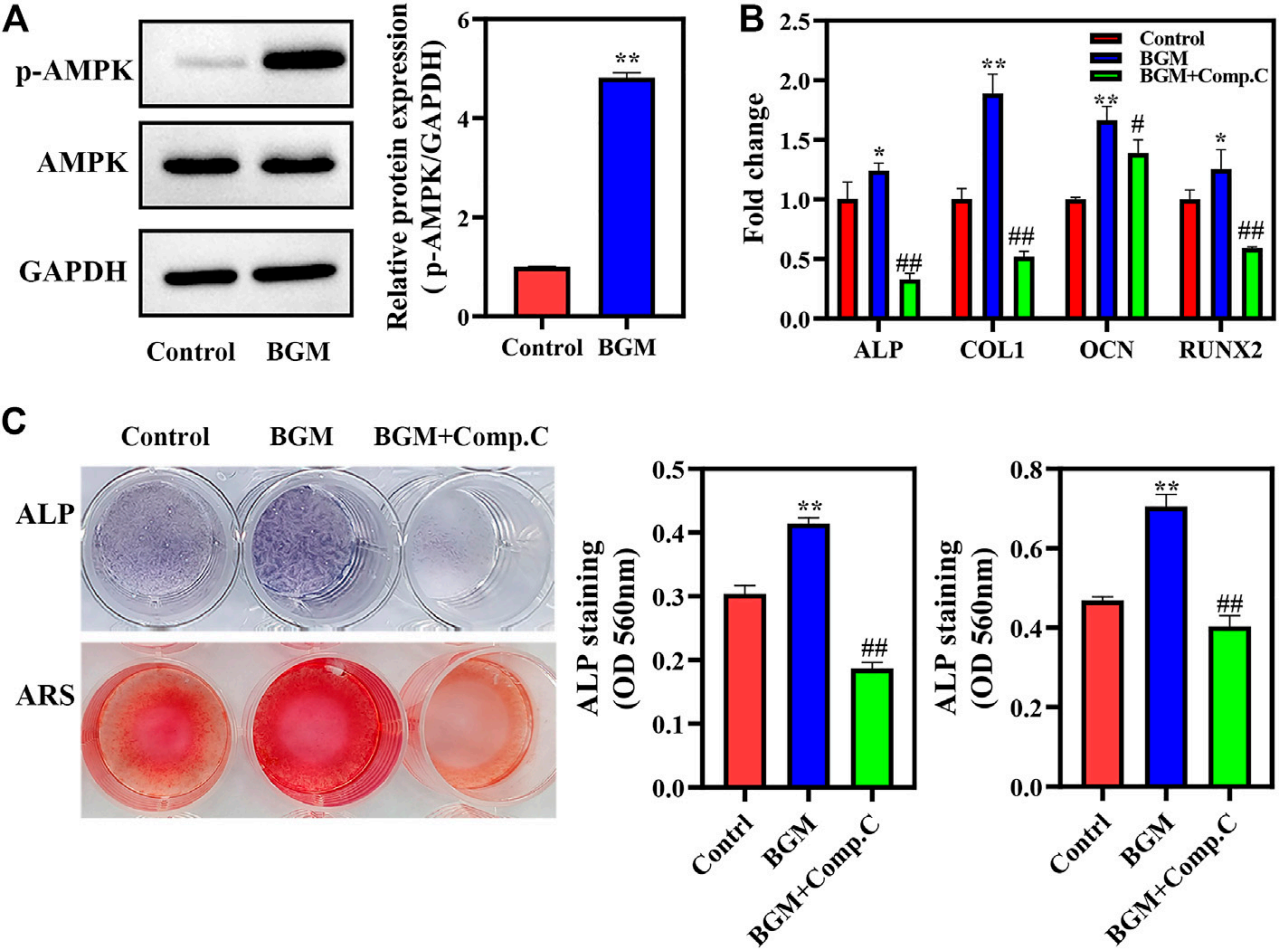
AMPK activation in BGM-stimulated osteogenic differentiation of SHED. **(A)** AMPKα phosphorylation was detected by western blot on day 4. The relative expressions were normalized against GAPDH. **(B)** mRNA expression of osteogenesis-related genes (ALP, RUNX2, OCN, and COL1) in SHED cultured for 7 days. **(C)** ALP, alizarin red staining and their semiquantification in SHED. *p < 0.05, **p < 0.01 versus the control group, ^#^ *p <* 0.05, ^##^ *p <* 0.01 versus the BGM group.

The mechanism by which BGM promotes osteogenesis was further studied. As shown in Figure 4A, BGM significantly promoted AMPK phosphorylation. A pharmacological inhibitor of AMPK (Compound C) significantly reduced BGM-upregulated ALP, RUNX2, OCN and COL1 mRNA levels (*p* < 0.05; Figure 4B). Additionally, ALP and alizarin red staining revealed that Compound C reduced the BGM-increased ALP activity and calcification, respectively (Figure 4C). These results suggest that AMPK signaling is involved in BGM-stimulated osteogenesis in SHED.

### *In vivo* bone regeneration

**FIGURE 5:**
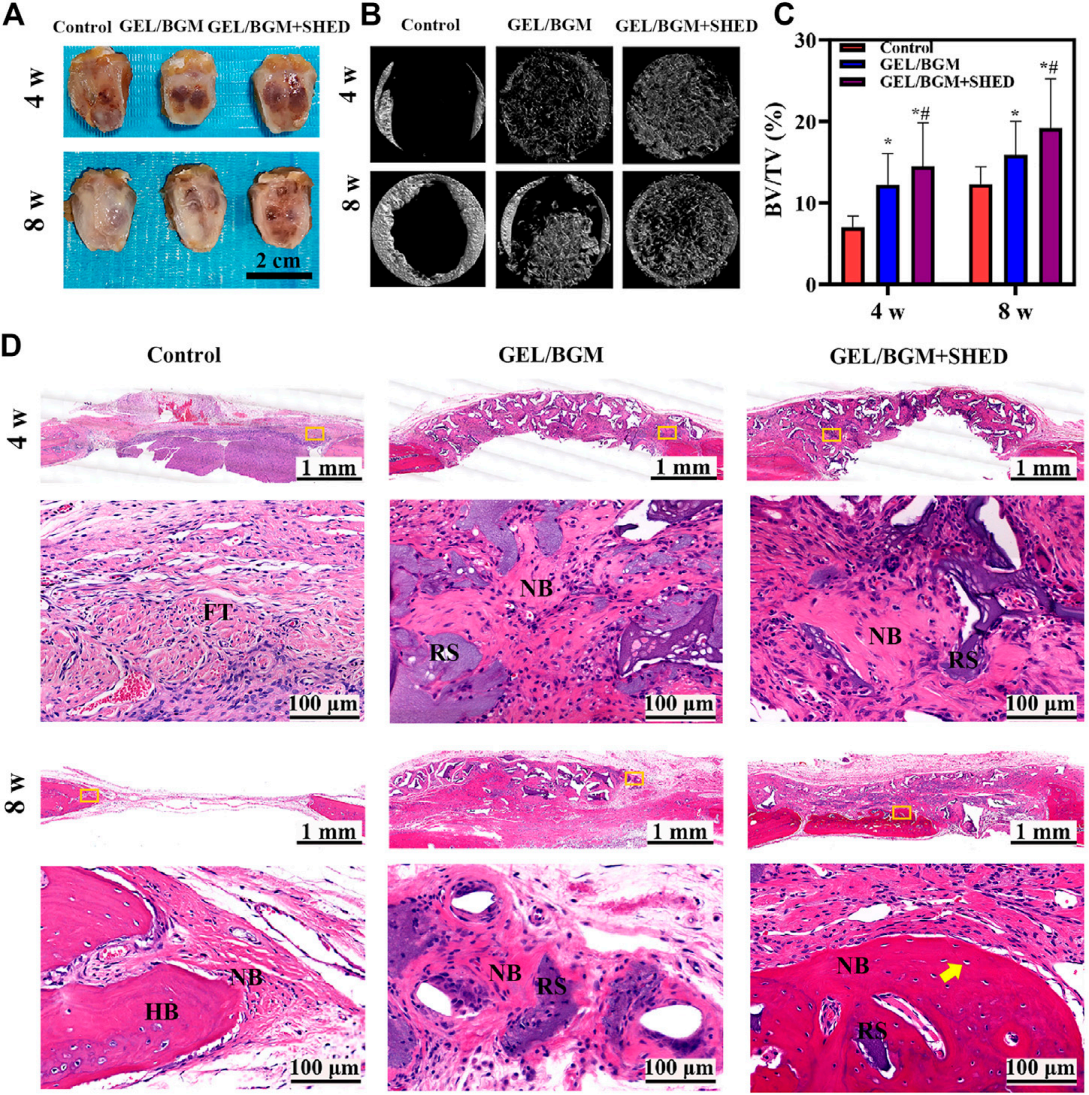
Evaluation of *in vivo* bone regeneration in rat cranial defects after implantation for 4 and 8 weeks. **(A)** Gross observation of the rat cranial defects; **(B)** Representative images of micro-CT in different groups; **(C)** Analysis of the bone volume to total volume (BV/TV) based on micro-CT results; **(D)** H&E histological images of the scaffolds with surrounding tissues. The first and third lines show the general view of the bone defect at 4 and 8 weeks, respectively; the second and fourth lines show the magnification of the yellow box in the first and third lines, respectively. Abbreviations are residual scaffolds (RS), host bone (HB), new bone (NB), fibrous tissue (FT) and osteocytes (yellow arrow). *p < 0.05 versus the control group, ^#^ *p <* 0.05 versus the GEL/BGM group.

At 4 or 8 weeks postimplantation, the calvarial bony defects were processed and analyzed. As shown in Figure 5A, the scaffolds maintained their original shape and were surrounded by soft tissue. Micro-CT was used to analyze the regenerated bone tissue (Figure 5B). After 4 weeks, limited new bone formed in the control group. In the GEL/BGM group, the defect was padded with scaffolds that provide support for bone regeneration. In the GEL/BGM + SHED group, in addition to the scaffolds, a few mineral deposits were visible. With time, newly formed minerals gradually occupied the defect area. Although there was no signal of complete healing, the GEL/BGM + SHED group had better healing than the control and GEL/BGM groups at 8 weeks postimplantation. To measure the quantity of newly formed minerals, bone volume to total bone volume (BV/TV) was used (Figure 5C). An increased number of mineral deposits was noted in the GEL/BGM and GEL/BGM + SHED groups compared with the control group (*p* < 0.05). The GEL/BGM + SHED group formed more new bone than the GEL/BGM group (*p* < 0.05).

An overview of representative histological sections of all groups is depicted in Figure 5D. When implanted *in vivo* for 4 weeks, only fibrous tissue regenerated without any bone formation in the control group, whereas new bone formation could be observed in the implant groups (GEL/BGM and GEL/BGM + SHED). Masson’s trichrome staining demonstrated that the GEL/BGM + SHED group regenerated more collagen fibers than the GEL/BGM group (Supplementary Figure S5C). A mild chronic inflammatory infiltrate was seen in all groups. After 8 weeks, the empty defect was mostly connected with loose fibrous connective tissue, and a limited amount of new bone formed at the margin of the defect area. In the GEL/BGM group, immature newly formed bones were observed not only at the edges of the bone defect but also dispersed into the remnant scaffolds. In the GEL/BGM + SHED group, mature newly formed bone tissues generated from the periphery to the central area of the defects. Lamellar organization with lacunae and osteocytes was observed in the new bone (yellow arrows, Figure 5D).

**FIGURE 6:**
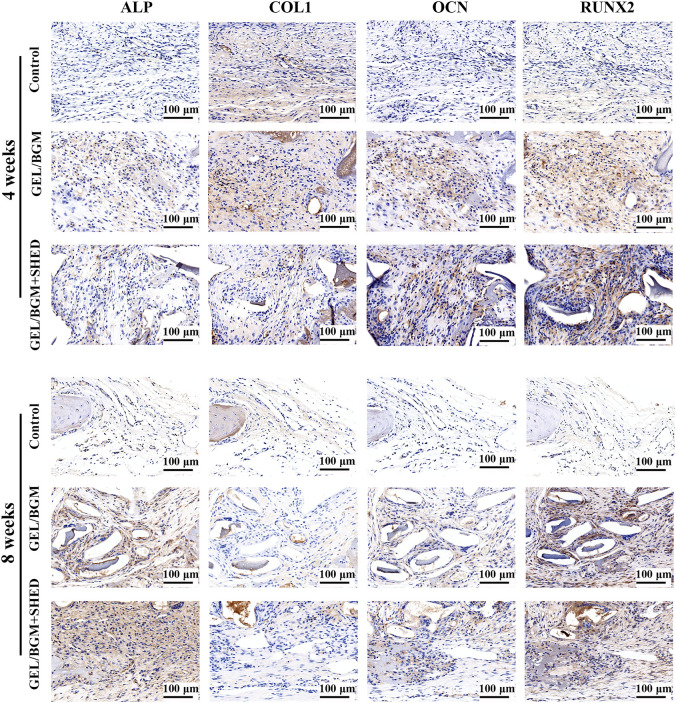
Immunohistochemical staining of ALP, COL1, OCN, and RUNX2 after implantation for 4 and 8 weeks.

**FIGURE 7:**
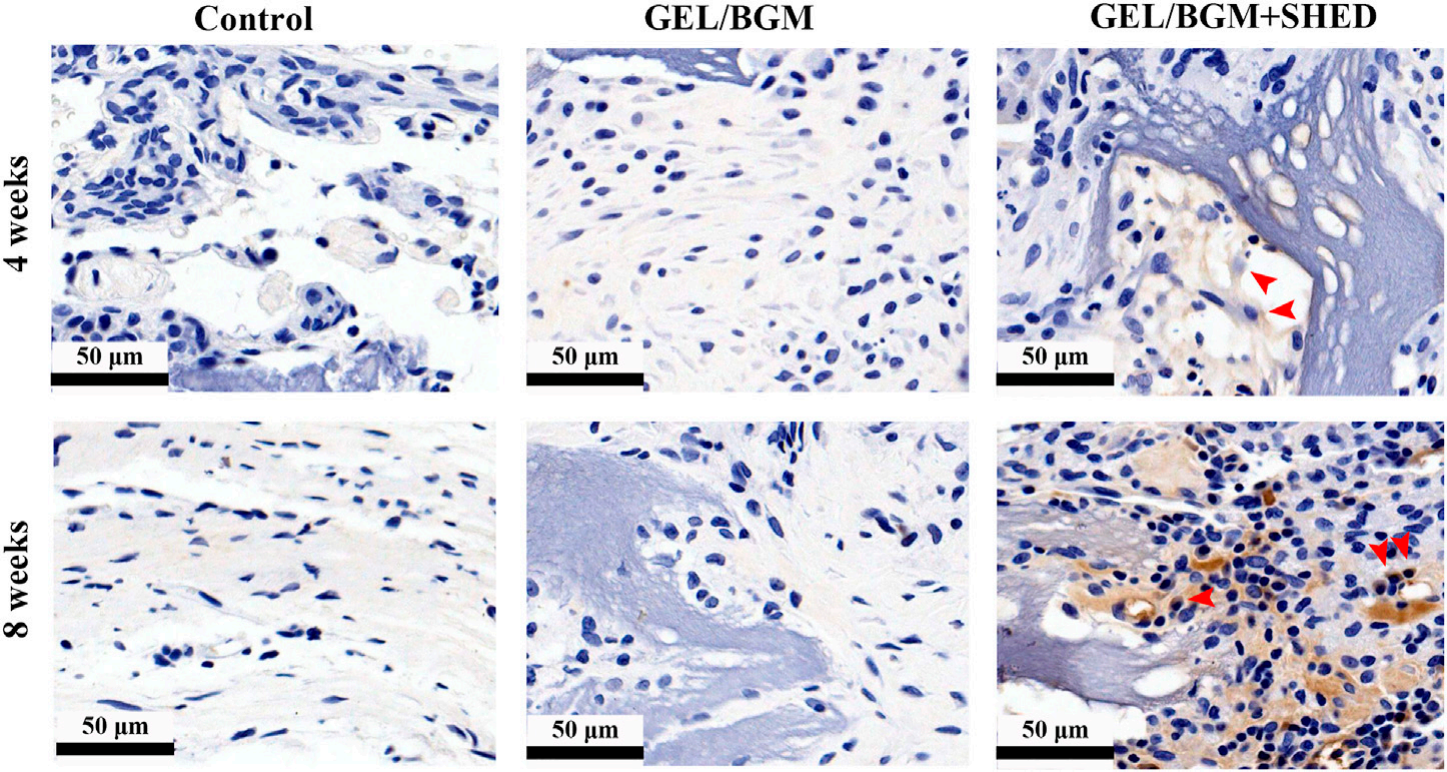
Immunostaining to detect SHED using human-specific anti-mitochondria antibody after implantation for 4 and 8 weeks.

The presence of osteogenic markers in the matrix of the new bone was analyzed by immunohistochemistry. After 4–8 weeks, faint staining was observed in the control group, indicating a lack of mature new bone formation in these samples. More positively stained area was found in the GEL/BGM + SHED group than in the GEL/BGM group (Figure 6; Supplementary Figure S6). Specific anti-human mitochondria antibody staining was used to detect the presence of SHED within the host tissues. As shown in Figure 7, appreciable persistence of SHED was observed around the scaffolds in the GEL/BGM + SHED group both 4–8 weeks after implantation (red arrows).

## Discussion

Repair of critical-size bone defects has long been a difficult challenge in regenerative medicine (Oryan et al., 2018a). The most critical cause for incomplete healing is that osteoprogenitors cannot migrate to the central portion of the defects (Freitas et al., 2019). In this study, we loaded SHED onto a GEL/BGM scaffold to construct a tissue engineering scaffold and found that it significantly accelerated the formation of new bone in critical-size bone defects.

SHED is a type of MSC with a strong proliferative capacity and multiple differentiation potentials (Miura et al., 2003). SHED can be easily obtained from naturally exfoliated deciduous teeth without invasive injury to children. MHC class II antigen HLA-DR binds to T-cell receptors during the immune response (Yang et al., 2021). SHED do not express HLA-DR even after stimulation with IFN-γ (Junior et al., 2020). With the properties of immunomodulatory functions, SHED is considered one of the most attractive cell sources for regenerative medicine (Sui et al., 2019). Immune rejection of xenogeneic cell transplantation is the most prominent obstacle for its clinical application, which negatively interferes with tissue repair due to a potentially more intense inflammatory process or even leads to transplant failure (Feng and Lengner, 2013). However, here, we implanted SHED in normal rats. SHED did not show more intense inflammatory infiltrate than the control or BGM groups, indicating its low immunogenicity. Another studies also reported that SHED incorporated into poly-(lactic-co-glycolic acid)-bioactive glass composite scaffolds or HA-beta TCP successfully regenerated new bones in rat calvarial bone defects with a mild chronic inflammatory infiltrate that were similar to that of the control groups (Kunwong et al., 2021; da Silva et al., 2022).

To avoid the easy loss of SHED in the bone defect sites, we loaded SHED into the GEL/BGM scaffolds. After implantation in a critical-size cranial defect model, the GEL/BGM + SHED group exhibited the best therapeutic effect among the three groups. Immunostaining of osteogenic-related proteins also confirmed the active regenerative processes of matrix deposition and calcification. SHED survived and proliferated on the scaffolds after implantation, as confirmed by the positive immunostaining for human mitochondria. Seo (Seo et al., 2008) found that human BSP and OC were positively detected on SHED transplants in mice, suggesting that SHED might directly differentiate into osteoblast-like cells and then secrete ECM. Another possible reason for its enhanced therapeutic effect might be that, as reported by Miura (Miura et al., 2003), SHED could stimulate the differentiation of recipient cells into osteogenic cells to generate new bone. SHED secrete some growth factors to accelerate the osteogenesis of host cells by paracrine action (Miura et al., 2003).

The rapid osteogenic differentiation of SHED is crucial for bone healing. Materials can affect the behaviors of stem cells (Li et al., 2015; Qiu et al., 2016). Enhanced ALP activity and mineralization formation as well as elevated osteogenic-related gene expression in SHED confirmed that BGM significantly augmented osteogenesis. Our results are consistent with other studies showing that BGM could stimulate the osteogenesis of other stem cells or osteoblast-like cells (Wang et al., 2016; Dittler et al., 2019). We further investigated the molecular mechanisms of BGM extracts on the osteogenesis of SHED. AMPK is a crucial sensor of cellular energy and nutrient status (Hardie, 2014). The AMPK pathway supports osteogenesis in MSCs (Wu et al., 2019; Kim et al., 2021). In this study, AMPK phosphorylation was significantly increased in BGM-treated SHED, while Compound C reduced the upregulation of osteogenic gene expression, ALP activity and mineral deposits induced by BGM, providing proof for the critical role of AMPK in BGM-stimulated osteogenesis of SHED.

## Conclusion

In this study, we easily obtained SHED and loaded them into GEL/BGM scaffolds to construct cell scaffolds. These GEL/BGM + SHED scaffolds exhibited low immunogenicity and significantly enhanced bone healing in critical-size cranial defects of rats. Mechanistically, BGM activated the AMPK signaling pathway in SHED. Therefore, GEL/BGM + SHED scaffolds represent a new promising strategy for critical-size bone healing.

## Data Availability

The original contributions presented in the study are included in the article/Supplementary Material, further inquiries can be directed to the corresponding author.
