# Supplementary material for: Easily attainable and low immunogenic stem cells from exfoliated deciduous teeth enhanced the in vivo bone regeneration ability of gelatin/bioactive glass microsphere composite scaffolds
Source: Front Bioeng Biotechnol. 2022 Dec 9;10:1049626. doi: 10.3389/fbioe.2022.1049626 (PMC9780285; doi:10.3389/fbioe.2022.1049626)
Supplement: Supplementary file 1 [file DataSheet1.docx]

**Supplementary Information**

**Supplementary figures**


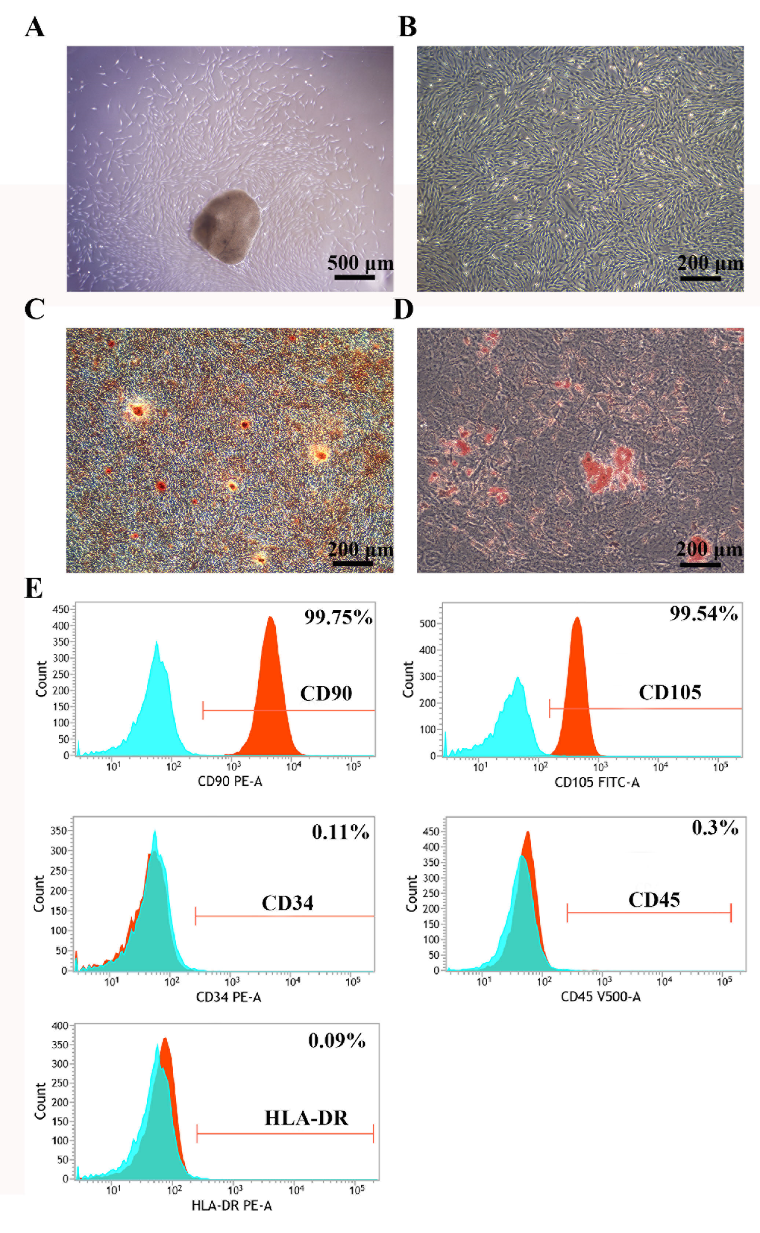


Fig. S1: Characterization of SHED. A: After pulp tissue culture for 3 days, spindle-shaped cells spread out in the culture flask. B: P2-generation cells were spindle shaped. C and D: Mineralized nodules and lipid droplets were found when SHED were cultured in osteogenic or adipogenic medium, respectively. E: Flow cytometric analyses showed that SHED were positive for CD90 and CD105 and negative for CD34, CD45 and HLA-DR.


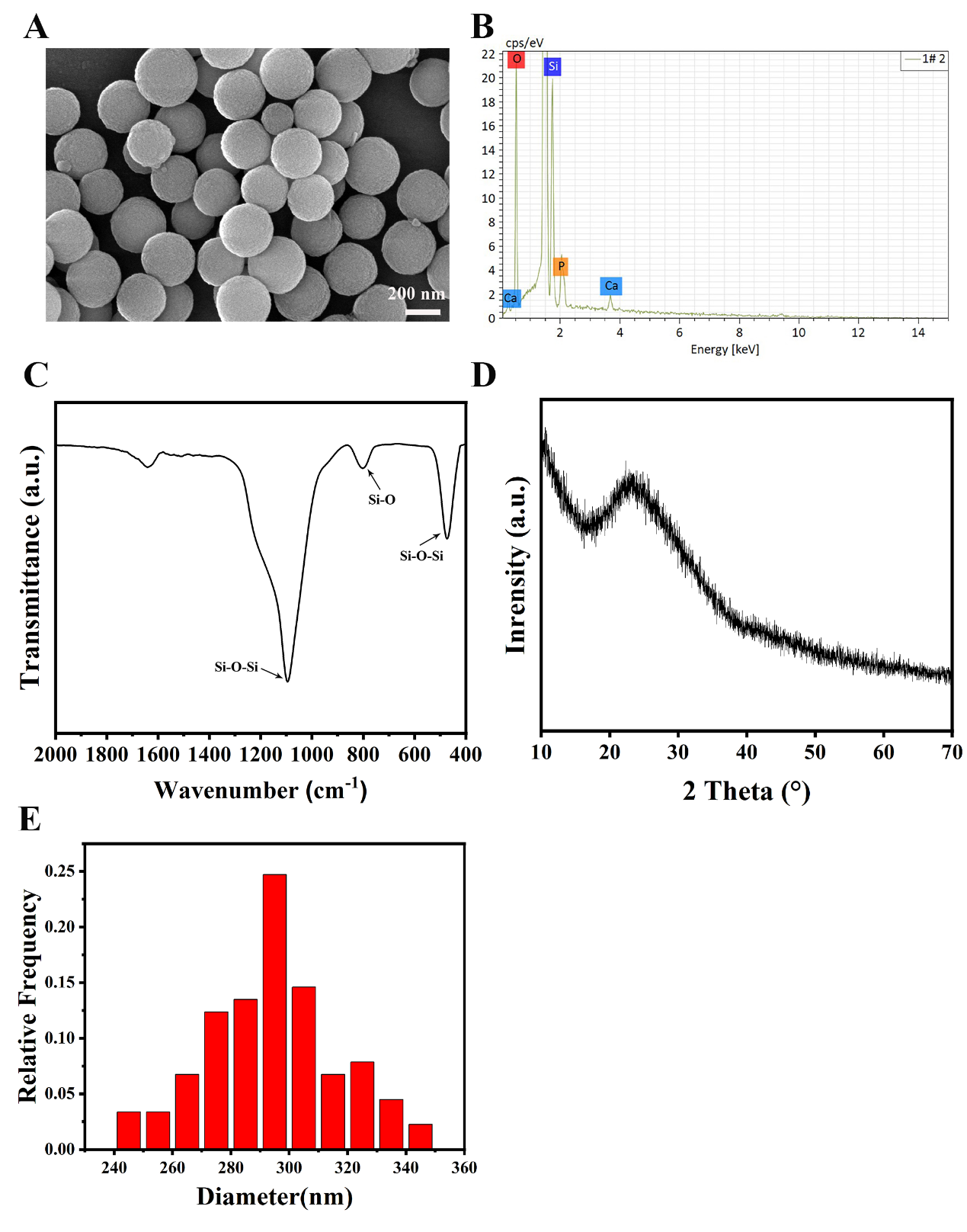


Fig. S2: Physicochemical structure characterizations of BGM. A: SEM image of BGM. B: EDS spectra of BGM. C: FTIR spectra of BGM. D: XRD pattern of BGM. E: Diameter distributions of BGM.


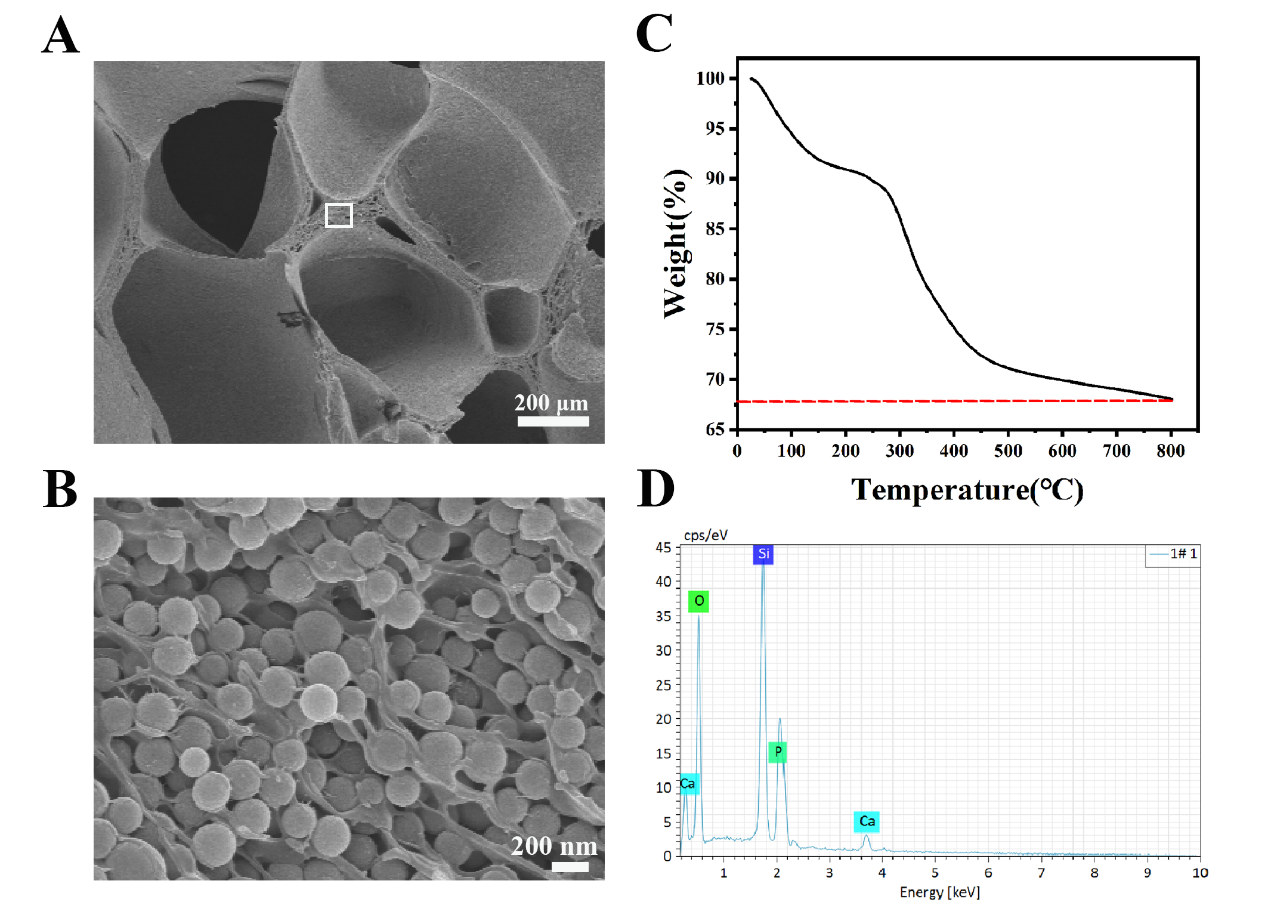


Fig. S3: Physicochemical structure characterizations of GEL/BGM scaffolds. A: SEM image of GEL/BGM. B: The magnification of the white box in figure A. C:TGA results of the scaffolds. D: EDS spectra of the scaffolds.


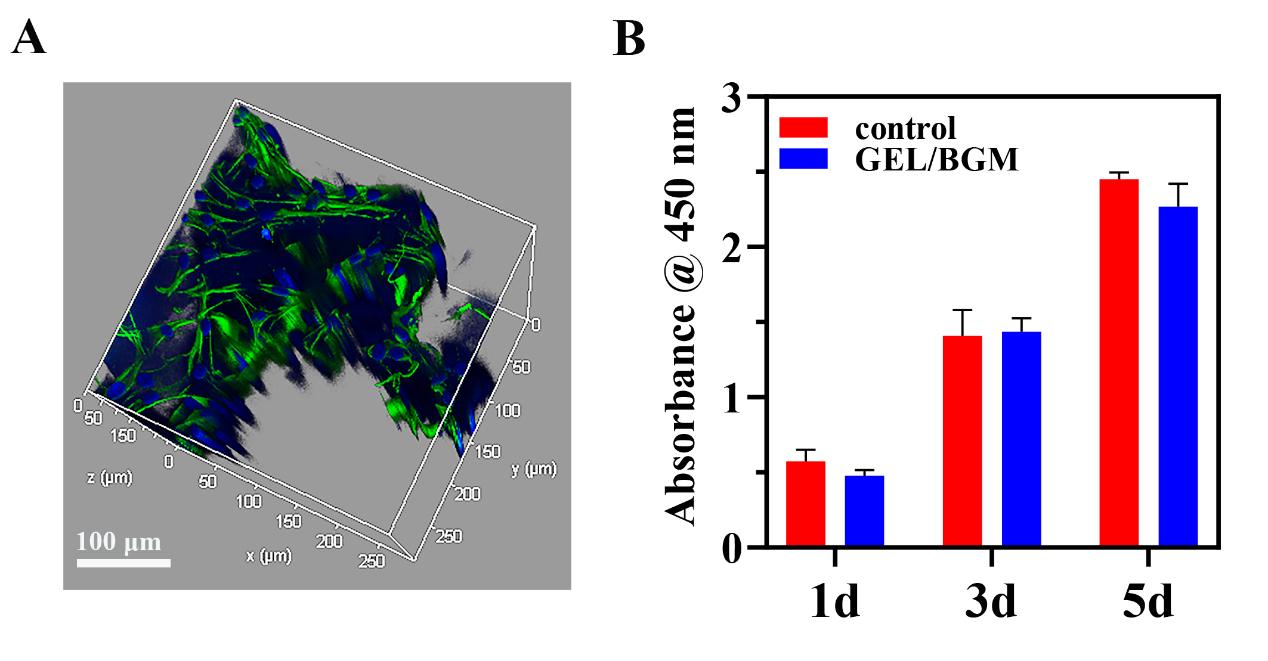


Fig. S4: Adhesion and proliferation of SHED on the GEL/BGM scaffolds. A: Confocal microscopy image of SHED adhered on the scaffolds. B: The proliferation of SHED after seeding on scaffolds for 1, 3 and 5 days.


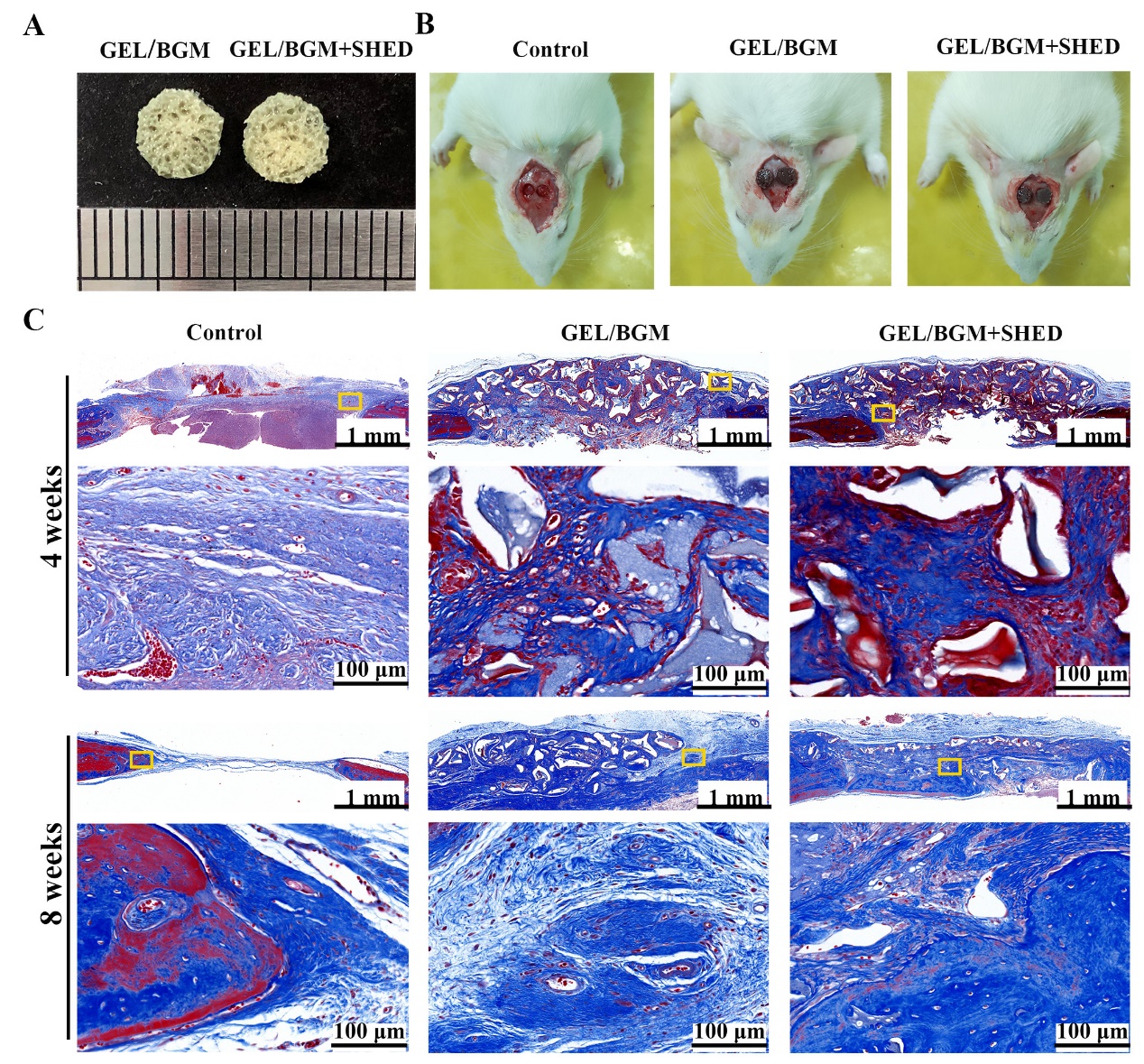


Fig. S5: Evaluation of in vivo bone regeneration in rat cranial defects. A: Gross observation of GEL/BGM and GEL/BGMBG+SHED scaffolds. B: Images of *in vivo* transplantation. C: Masson's trichrome staining of the scaffolds around tissues. The first and third lines show the general view of the bone defect at 4 and 8 weeks, respectively; the second and fourth lines show the magnification of the yellow box in the first and third lines, respectively.





Fig. S6: Semiquantitative analysis of immunohistochemical staining of ALP, COL1, OCN and Runx2 at 4 and 8 weeks. ^*^*P <* 0.05 versus the control group, ^#^*P <* 0.05 versus the GEL/BGM group.

**Table S1.** qRT-PCR Primer Sequences

| Gene | Primer sequence |
| --- | --- |
| ALP | Forward: 5’-AAGGACATCGCCTACCAGCTC-3’  Reverse: 5’-TCTTCCAGGTGTCAACGAGGT-3’ |
| Runx2 | Forward: 5’-TGGTTACTGTCATGGCGGGTA-3’  Reverse: 5’-TCTCAGATCGTTGAACCTTGCTA-3’ |
| OCN  COL1 | Forward: 5’-GGACTGTGACGAGTTGGCTGAC-3’  Reverse: 5’-TGCCTGGAGAGGAGCAGAACTG-3’  Forward: 5’- CCCTGGAAAGAATGGAGATGAT-3’  Reverse: 5’- ACTGAAACCTCTGTGTCCCTTCA-3’ |
| GAPDH | Forward: 5’-CCTGGATACCGCAGCTAGGA-3’  Reverse: 5’-GCGGCGCAATACGAATGCCCC-3’ |
